# Adjuvant Cardioprotection in Cardiac Surgery: Update

**DOI:** 10.1155/2014/808096

**Published:** 2014-08-19

**Authors:** Robert Wagner, Pavel Piler, Zufar Gabbasov, Junko Maruyama, Kazuo Maruyama, Jiri Nicovsky, Peter Kruzliak

**Affiliations:** ^1^Department of Cardiovascular Anesthesiology, Centre of Cardiovascular and Transplant Surgery, Pekarska 53, 656 91 Brno, Czech Republic; ^2^Department of Cardiovascular Diseases, International Clinical Research Center, St. Anne's University Hospital and Masaryk University, Pekarska 53, 656 91 Brno, Czech Republic; ^3^Department of Cardiovascular Surgery, Centre of Cardiovascular and Transplant Surgery, Pekarska 53, 656 91 Brno, Czech Republic; ^4^Institute of Experimental Cardiology, Russian Cardiology and Research Complex, 3rd Cherepkovskaya 15-A, 12552 Moscow, Russia; ^5^Department of Anesthesiology and Critical Care Medicine, Mie University School of Medicine, 1577 Kurimamachiya-cho, Tsu City, Mie Prefecture 514-8507, Japan; ^6^Department of Clinical Engineering, Suzuka University of Medical Science, 1001-1 Kishiokacho, Suzuka, Mie Prefecture 510-0226, Japan

## Abstract

Cardiac surgery patients are now more risky in terms of age, comorbidities, and the need for complex procedures. It brings about reperfusion injury, which leads to dysfunction and/or loss of part of the myocardium. These groups of patients have a higher incidence of postoperative complications and mortality. One way of augmenting intraoperative myocardial protection is the phenomenon of myocardial conditioning, elicited with brief nonlethal episodes of ischaemia-reperfusion. In addition, drugs are being tested that mimic ischaemic conditioning. Such cardioprotective techniques are mainly focused on reperfusion injury, a complex response of the organism to the restoration of coronary blood flow in ischaemic tissue, which can lead to cell death. Extensive research over the last three decades has revealed the basic mechanisms of reperfusion injury and myocardial conditioning, suggesting its therapeutic potential. But despite the enormous efforts that have been expended in preclinical studies, almost all cardioprotective therapies have failed in the third phase of clinical trials. One reason is that evolutionary young cellular mechanisms of protection against oxygen handling are not very robust. Ischaemic conditioning, which is among these, is also limited by this. At present, the prevailing belief is that such options of treatment exist, but their full employment will not occur until subquestions and methodological issues with the transfer into clinical practice have been resolved.

## 1. Introduction

The spectrum of cardiac patients has recently shifted to groups exposed to a higher risk level in terms of age and comorbidities, as well as the type of treatments needed. This increases the need for emergency surgery in acute coronary syndromes with complications including acute heart failure [1]. Another growing group of patients comprises those with advanced chronic heart failure who require long-term, combined treatment. Similarly, a longer graft ischaemia is often needed in heart transplantations. These groups of patients have a higher incidence of postoperative complications (acute heart/renal failure, cerebral stroke) and ultimately a higher mortality. One factor to consider involves the current limits for perioperative myocardial protection [2, 3]. Some patients may be offered revascularisation on the beating heart, transcatheter implantation of heart valve prosthesis, or a mitral clip, but for the surgical field to be peaceful and bloodless, the majority of high-risk patients are operated on using the so-called ischaemic cardioplegic arrest. Here, the restoration of the coronary circulation is accompanied by acute ischaemia-reperfusion injury (IRI) with raised cardiac enzymes [4]. Some degree of cardiac necrosis is inherent in each cardiac surgery and, in addition to reperfusion injury, multiple factors may be involved [5]. According to recent studies, the incidence of myocardial infarction (MI) after CABG ranges, depending on the definition, from 2% to 10% [6]. According to the latest revised definition, MI arising in connection with CABG (“Category 5”) is arbitrarily determined by a 10-fold increase in cardiac-specific enzymes during the first 48 hours along with ECG signs of necrosis or displaying a coronary occlusion/contractility disorder [7, 8]. The term “perioperative myocardial injury” describes a condition that, although not fully achieving MI type 5, has health consequences even at this level of affection. A new retrospective study on 18,908 CABG patients has found that CK-MB/troponin elevations in the initial 24 hours were associated with increased mortality in the coming months to years [9]. Thus, it is obvious that the restriction of perioperative myocardial injury is important for the prognosis of the patient. The facts mentioned above open the door to finding other methods of perioperative myocardial protection in cardiac surgery.

## 2. Cardioplegia

The current gold standard of cardioplegia is a depolarisation myocardial arrest through perfusion of the coronary arteries using a hyperkalaemic solution. The time of reversible ischaemia that it provides is satisfactory for the surgery (up to 4 hours); plus, there is the restoration of function (a number of days) and low systemic toxicity [10]. Myocardial protection is expressed by delaying irreversible ischaemia, to which the arrest of electrical and mechanical activity is a contributing factor. The use of potassium, however, is not lacking in problems; in addition to the very narrow safety profile of extracellular potassium (10–30 mmol per litre), there is a calcium overload of the myocytes; plus, there are other types of ionic imbalance that lead to arrhythmias and depressed myocardial function persisting over several days [11]. Myocardial protection is reinforced using hypothermia, mixing the cardioplegic solution with the patient's blood and a number of additives: procaine, adenosine (augmented attenuation of electrical activity), calcium antagonists, magnesium (inhibition of calcium overloading), desensitisation of calcium channels (inhibition of calcium at the myofilament level), energy substrate (anaerobic ATP production), mannitol, Fe chelates (oedematous and oxidation control effect), and others. Additives are not part of every cardioplegic solution, because they are accompanied by side effects [12]. An alternative to the hyperkalaemic depolarisation arrest is the hyperpolarisation arrest induced by decreasing sodium and calcium from solutions such as Bretschneider solution and its later version, Custodiol HTK (histidine-tryptophan-ketoglutarate solution), but even this approach does not lead to a qualitative change in myocardial protection from reperfusion injury [2]. Hyperpolarisation can also be induced by agents that open membrane potassium channels (outflow of potassium from myocytes). This leads to the reduction in the calcium influx into myocytes (by reducing the action potential) without having to add other agents with adverse effects. Studies with aprikalim, pinacidil, or nicorandil showed a comparable or better cardioprotection level but failed in clinical practice due to a long elimination time and systemic hypotension [13].

## 3. Reperfusion Injury (RI) of Myocardium

Myocardial RI is a complex response of the organism to the restoration of coronary blood flow in the ischaemic tissue and is an important component of ischaemia-reperfusion injury [14, 15]. The flow restoration rescues viable myocytes, accelerating the formation of necrosis of irreversibly damaged cells [16, 17]. In another portion of reperfused myocardium, there are subtle changes such as cell swelling, enlargement of mitochondria, or loss of myofibrillar organisation. The exposed area may recover or reach up to the death of cells, the morphological correlates of which are, at the tissue level and organ levels, referred to as contraction band necrosis [18] and the no-reflow phenomenon [19], respectively. This observation led to the concept of lethal reperfusion injury as early as 1985 [20]. RI is mostly manifest in reversible changes such as ventricular arrhythmias [21] and contractile dysfunctions (stunning) [22, 23]. The restoration of coronary flow after cardioplegic arrest is also manifest in disorders of rhythm and contractility, with contraction band necrosis possibly found in the myocardium. These were detected in 26% of early deaths of CABG patients, whilst also being found in almost all those with synchronous myocardial infarction as a result of graft occlusion [24].

Although the no-reflow phenomenon was described as early as 1966, the aetiology is still unclear. There are a number of other events involved, including embolisation of debris from the site of the occlusion, the release of vasoconstrictor and thrombogenic factors, and inflammatory substances; plus there are considerations in respect of the structural collapse of the capillary bed [25]. Whether RI is an independent factor responsible for additional tissue necrosis or simply speeds up exiting of cells condemned to death from ischaemia has been the subject of debates, the process being most intense in the 1990s [26], since it is difficult to discern the death of myocytes that were viable at the end of ischaemia. However, it is now accepted that RI is an independent factor for the spread of infarction after myocardial ischaemia [27]. The evidence is, nonetheless, only indirect, relying on the positive impact of therapeutic interventions at the reperfusion stage. Cardiac surgery has long been aware of the positive effect of the modified reperfusion (temperature and composition) and staged reperfusion (a slow, 20-minute restoration of coronary flow) [28, 29]. Mainly, the 36% reduction in infarction in STEMI patients treated with primary PCI and randomized to ischaemic postconditioning (see below [30]) is indirect evidence of this.

### 3.1. The RI Mechanisms

Oxygen is the main factor of damage, that is, its acute lack in the phase of ischaemia and toxicity in the reperfusion phase. This paradox can be explained by the evolution of the relationship between organisms and oxygen on Earth. After the increase in concentration of oxygen in the atmosphere 2.4 billion years ago that nearly wiped out life on Earth (the great oxygenation event), aerobic organisms entered the path of adaptation, eventually ending up on oxygen, an electron acceptor for their energy mechanisms. Multicellular organisms thus gained the opportunity of explosive development at the cost of shortening the lifespan of individuals. Although widespread, the evolutionary young cellular mechanisms of protection against oxygen handling are not very robust. To this day, eukaryotic cells are dealing with a difficult logistical task, that is, to bring enough oxygen to the cell for respiration whilst eliminating the pernicious consequences of its presence. The start of reperfusion is the moment at which cells are most vulnerable, and as for antioxidant enzymes to regenerate, available energy is needed [31, 32].

**Figure 1:**
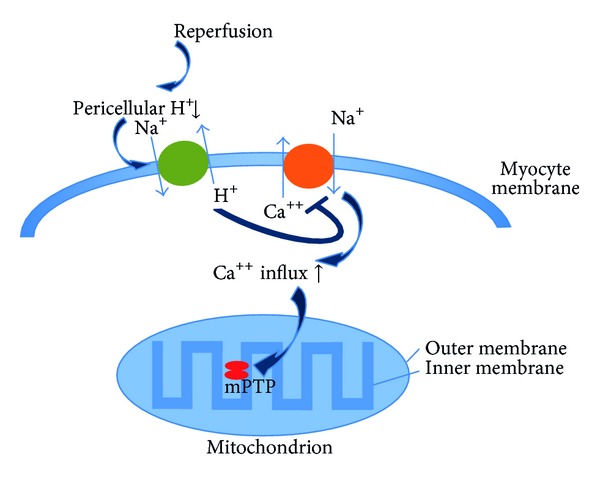
Role of mitochondrial permeability transition pore (mPTP) in myocardial reperfusion injury.

The closure of the coronary artery leads to a series of changes, which begins with dysfunction and ends with myocardial infarction. The changes mainly concern the reduction of energy production and consumption. From the onset of ischaemia in myocytes, there is a sharp ongoing decline in ATP, with anaerobic utilisation of energy with a subsequent decrease in pH. Acidosis leads to contractility arrest within minutes, and complete depletion of ATP over 15–30 minutes results in myocyte death, at least as part of experiments. Intracellular acidosis is mitigated by a sodium-hydrogen pump; this taking place at the cost of intracellular entry of sodium, then water, and then even calcium. Deficient membrane ATPases are at the beginning of the disorder of the control of intracellular calcium, which leads to the first calcium overload. The hypothesis of ATP depletion being a central cause of ischaemic death still applies [33]; it was, however, extended by subsequent damage in the reperfusion phase. The restored oxygen supply and energy production in a situation of abnormal cellular environment leads to further damage [34]. Endogenous defence mechanisms are also activated in the early phase of reperfusion; these designed to minimize further damage to the myocardium; more specifically, there is a decision-making process as to which myocytes will be repaired or eliminated by apoptosis. Interconnected pathophysiological processes are supportive of RI, such as rapid pH fluctuations (pH paradox), oxygen toxicity (oxygen paradox), calcium overload of cells (calcium paradox), and inflammation [35]. Reperfusion promptly washes off the low pericellular pH with the emergence of a large H^+^ gradient on myocyte membranes. Activation of the Na/H pump follows, as well as the rapid entry of sodium into myocytes. This causes a passive reverse running of the membrane Na/Ca exchanger, the exchange of sodium for calcium causing the second intracellular calcium peak. The result of the rapid pH adjustment and calcium overload comprises an abolition of protease disinhibition (calpain, etc.), opening of mitochondrial transport channels (mPTP) and hypercontracture of the myofibrils (Figure 1). Reoxygenation starts aerobic ATP production, accompanied by explosive formation of the reactive oxygen species (ROS), the main sources of which involve calcium-activated xanthine oxidase and cytochrome of the respiratory chain. After overcoming the capacity of the main antioxidation enzymes SOD (superoxide dismutase) and catalase, the excess of ROS damages cell structures, especially membrane proteins and phospholipids. However, the initial amount of ROS during ischaemia alone and even at the beginning of reperfusion is necessary as a signal that activates defence mechanisms. These processes, along with the activation of inflammation cascades and bioactive factors such as cytokines, may lead to death of myocytes [36]. In the best case, the gradual regulation of ionic and electrophysiological processes in membranes is accompanied by arrhythmias and contractile dysfunction [37].

## 4. Myocardial Conditioning (MC)

One of the new ways of augmenting intraoperative myocardial protection is the activation of an innate defence mechanism to avoid reperfusion injury; one that was termed myocardial conditioning. Originally discovered in 1986 in the analysis of cumulative episodes of coronary occlusion and reperfusion, this principle was termed ischaemic preconditioning. Murry et al. described, on a canine model, a phenomenon in which four five-minute cycles of coronary occlusion and reperfusion prior to the sustained 40-minute occlusion reduce the extent of infarction by 25% [38]. This definition was further enhanced by the knowledge that ischaemic preconditioning also reduces dysrhythmias and myocardial dysfunction [39, 40]. The discovery was preceded by finding a warm-up phenomenon: patients with prodromal angina pectoris often show minor variations in the ST segment, less myocardial dysfunction, and an even lesser extent of necrosis [41]. Ischaemic preconditioning is underway in two stages, the early stage beginning immediately after ischaemia/reperfusion stimuli and lasting up to three hours, and is followed by a 12- to 24-hour interval after which there is the onset of the late stage; one that is weaker but lasts three days [42]. The early stage depends on the activation of available signalling molecules, receptors, and intracellular pathways, while the late stage requires the expression of defensive genes and de novo protein synthesis; see below. Myocardial conditioning evolved into multiple modalities that may be applied before (preconditioning), during (perconditioning) and immediately after the ischaemic insult or at reperfusion (postconditioning). The stimulus can be applied directly to the myocardium or a remote tissue (remote conditioning).

### 4.1. Molecular Mechanisms of MC

Despite the accumulation of facts as regards the mechanism of inception, spreading, and implementation of the cardioprotective signal, there is now some sort of consensus as to the architecture of these; it can be divided into three levels (triggers, intracellular pathways, and end-effectors) [43]. Not only ischaemic, but also other types of stress (thermal, chemical), release triggers from the autocrine source (adenosine) or exogenous/paracrine sources (bradykinin, opioids). The triggers bind to receptors of the myocyte membrane, followed by a cascade activation of proteinases of multiple parallel pathways, with this ending at the effector (mitochondria, cytoskeleton).

Intracellular signal transmission takes place via at least three parallel channels: the first is activated by receptors coupled with G protein (GPCR) and proceeds via a nitric oxide (NO), cGMP, and PKG (protein kinase G). The second channel, also activated by GPCR and termed RISK (reperfusion injury salvage kinase), contains a number of kinases including PKB (protein kinase B), ERK (extracellular regulated kinase) and key GSK 3beta (glucose synthase kinase). The third channel is SAFE (survival activating factor enhancement), which is activated by TNF-alpha and includes JAK (Janus kinase signal transducer) and STAT3 (mitochondrial activator of transcription).

**Figure 2:**
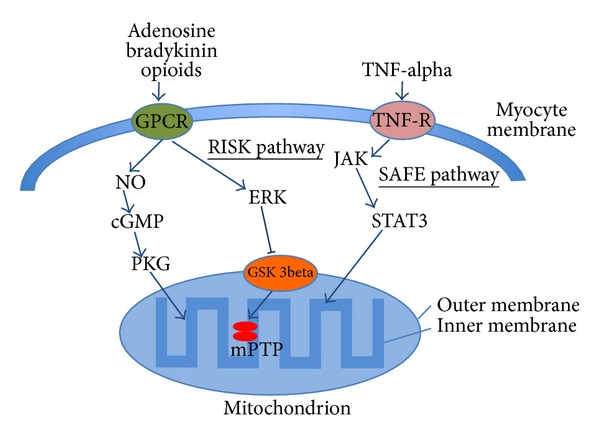
Molecular mechanism of myocardial conditioning. cGMP, cyclic guanosine monophosphate; ERK, extracellular regulatory kinase; GSK 3beta, glucose synthase kinase 3 beta; GPCR, G-protein-coupled receptor; JAK, Janus kinase signal transducer; mPTP, mitochondrial permeability transition pore; NO, nitric oxide; PKG, protein kinase G; RISK, reperfusion injury salvage kinase; SAFE, survival activating factor enhancement; STAT3, signal transducer and activator of transcription 3; TNF-alpha, tumour necrosis factor-alpha; TNF-R, TNF receptor.

Mitochondria are considered key effectors of cardioprotection, with mPTP (mitochondrial permeability transition pore) being the primary end point. They are nonspecific channels in the inner mitochondrial membrane, their physiological role being not known in detail [44]. Ischaemic stress opens mPTP, penetration of ions and water leads to swelling as far as rupture of the outer membrane, the release of proteins including cytochrome c, caspase activation, and apoptosis of the cell. Inhibition of GSK 3beta is an integration point of activation of protein kinase pathways, and, along with Connexin 43 and activation of potassium ATP channels (KAPT), “holds” mPTP in the closed state during the critical phase of reperfusion. Mitochondria are also a source of cardioprotective signal. At the start of reperfusion, it is necessary to produce a certain amount of reactive oxygen species that amplify protective mechanisms. Important factors also include the presence of acidosis, which is involved in the closure of mPTP and inhibits excessive contractile activity in the presence of excess calcium ions. The immediate reperfusion is thus a crucial moment when protective mechanisms (kinase signalling systems, ROS, acidosis) can be not only activated spontaneously or enhanced by the ischaemic pre/postconditioning, but also violated by inappropriate interventions, for example, incorrect timing of alkalising substances and antioxidating agents.  Figure 2 summarizes the molecular mechanisms of MC.

### 4.2. The Late Stage of Cardioprotection

The mechanisms of the acute and late stages of cardioprotection have many things in common. Some mediators of the intracellular signal transduction, however, induce gene transcription and synthesis of defensive proteins within 12–24 hours after the ischaemic stimulus [45]. These include transcription factors JAK-STAT 1/3 (Janus kinase/signal transducer and activator of transcription), PKC (protein kinase C), NF-kappa B (nuclear factor-kappa B), AP-1 (activator protein-1) and HIF-1 alpha (hypoxia inducible factor-1). This is followed by the synthesis of iNOS (induced nitric oxide synthases), Cox-2 (cyclooxygenase type 2), aldose reductase, mSOD (mitochondrial superoxide dismutase), and HSP (heat shock proteins). The products of these enzymes directly affect the mPTP as NO [46] and regulate the excessive production of ROS and aldehydes [47, 48]; plus, they protect the structure of proteins using HSP [49]. The detailed task of Cox-2 products (prostaglandins: PGE2, PGF1-alpha) is not yet known [50].

### 4.3. Ischaemic Postconditioning

Another modality which can reduce the size of myocardial infarction involves the application of several ischaemia-reperfusion stimuli immediately (within the first minute) after the restoration of perfusion in the ischaemic region [51]. The reduction in infarction was observed in all species of tested animals [52] and also in a clinical trial [30]. The mechanism is, in many respects, the same as for the standard modality, both of them sharing the necessity of the reperfusion stage, but here the SAFE kinase pathway dominates over RISK. While ischaemic preconditioning may be used only for elective procedures (percutaneous or surgical), postconditioning can be applied even to patients during primary percutaneous coronary interventions (PPCI).

### 4.4. Remote Ischaemic Conditioning: RIPC

Remote ischaemic conditioning is a form of cardioprotection induced by short cycles of ischaemia and reperfusion, which are applied to a distant tissue and/or organ. The phenomenon was originally observed intraorganally and it is interesting that it was predicted using a mathematical model [53]. In the original experiment, four cycles of five-minute occlusion-reperfusion in the circumflex branch of the left coronary artery led to a reduction of infarction in the area of ramus interventricularis anterior, which was subjected to 60-minute occlusion [54]. Subsequent studies showed that cardioprotection can be achieved even by applications to remote organs like kidney, intestine, brain, and skeletal muscle [55]. Remote pulses can be applied during an ongoing ischaemia (remote perconditioning), which can be advantageous prior to reception in STEMI patients [56] and also at the beginning of reperfusion (remote postconditioning) [57].

The mechanism of RIPC at the myocardial level is largely the same as in the basic application, but the transfer of the protective signal from a remote organ to the myocardium is not fully explained. Three channels of communication were designed and partially tested: the humoral blood channel, neuronal stimulation, and communication of immunity cells. The humoral mechanism was tested by a perfusate and dialysate from the ischaemic organ, which induced cardioprotection after being applied to the isolated myocardium [58]. The identity of humoral factors also remains speculative. Classic triggers (bradykinin, opioids), but also as yet undefined small hydrophobic molecules, are considered [59]. Neuronal stimulation was verified by stimulation of the femoral nerve with the conclusion that intact neural pathways are required for the release of humoral components [60]. Other studies focused on parasympathetic activity, concluding that RIPC is dependent on the activity of specific vagal preganglionic neurons [61]. Not surprisingly, many assume the necessary interplay of these components [62].

Its noninvasiveness and ease of application determined RIPC to be the most tested modality in clinical research. In terms of orders, dozens of “proof of concept” clinical studies have tested remote conditioning by an application of ischaemia/reperfusion on the upper/lower limb using a pressure cuff in elective operations in cardiac surgery and invasive cardiology [63–71]. Nonetheless, not every study has confirmed cardioprotection [72–74].

## 5. Clinical Studies

The basic type, ischaemic preconditioning (IPC), was first used in clinical testing on a small set of CABG patients in 1993, the stimulus comprising repeated aortic clamping and declamping prior to the cardioplegic arrest itself. The IPC group was observed to have an increased level of ATP in biopsy samples and lower levels of serum troponin I [75, 76]. Meta-analysis of 22 similar studies (937 patients) found fewer arrhythmias, a lower consumption of inotropes, and shorter stay in the ICU in the IPC group, but these were not the main parameters assessed [77]. This basic technique was, however, not developed any further due to its invasive nature (risk of thromboembolism in the handling of the aorta) and the need for extended surgery time.

Remote ischaemic conditioning (RIC) has regained interest in clinical testing. The first clinical trial was performed on a group of 37 children undergoing surgery for congenital heart defect. RIC stimulus comprised three five-minute inflations/deflations using a pressure cuff (200 mmHg) on the lower limb before connecting to extracorporeal circulation. Children randomized in the RIC group had a lower consumption of inotropes, lower inspiratory pressures, and lower serum troponin I concentrations for 24 hours after the surgery [78]. A series of similar studies followed, mainly in operations for CHD, but differences in the assessed parameters were not always found [72, 79, 80]. The reasons for the negative results could involve issues in transferring the experimental results into clinical practice in general (see below) and also differences in protocols (stimulus magnitude and timing), the selection of patients (age, comorbidities, and extent and type of operation), use of anaesthesia (IV versus inhaled anaesthetics), and others. A new meta-analysis of clinical studies on CABG patients, however, revealed that RIC reduced perioperative myocardial injury as measured by lower levels of serum troponin [81]. Currently ongoing large multicenter studies (ERICCA, RipHeart) are expected to resolve the issues [82].

Ischaemic postconditioning (IPostC) has been successfully tested in paediatric cardiac surgery. The IPostC stimulus comprised repeated 30-second aortic declamping and clamping before the definite myocardial reperfusion. Children randomized in the IPostC group had lower serum levels of CKMB and troponin T for two hours after the surgery [83]. Use in adults has the disadvantage of increased risk of thromboembolic complications in handling the aorta [84]. The children's cardiac surgery also tested the remote application of the stimulus (remote ischaemic postconditioning, again with a lower release of troponin [85]), but again there was a failure of clinical effect in a study on 1,280 patients [86]. The authors themselves admitted that the propofol (a scavenger of oxygen radicals) that was used could void the cardioprotective effect of ischaemic preconditioning as demonstrated in previous studies [87]. IPostC was also clinically tested in invasive cardiology in primary percutaneous interventions, and, as already mentioned, the positive results of this study became indirect proof of the existence of reperfusion injury [30]. Checking of the usefulness in this indication is foreseen in the ongoing large multicenter studies. The initial results suggest that IPostC and other techniques benefit mainly STEMI patients with anterior localisation as well as a greater extent of affection [56].

Compared to the previous modalities, the late phase of ischaemic preconditioning was clinically tested only in two studies. Our research group conducted a study based on some negative studies in CABG patients. At the start of surgery or, more specifically, prior to the cardioplegic cardiac arrest itself, there are stress stimuli that may also activate the phenomenon of the early stage of ischaemic preconditioning (skin incision, sternotomy, and cardiopulmonary bypass) and the IC stimulus applied, local or remote, already comes as an extra event [88]. In addition, the early IC stage lasts a maximum of three hours, which does not even cover the period of surgery, not to mention the initial hours after the surgery, when there is the highest frequency of complications. In contrast, the late IC stage takes up to three days and offers protection from adverse events even in the early postoperative period. Our study was conducted on 60 CABG patients, the remote IC stimulus comprising three five-minute inflations/deflations using a pressure cuff applied to the upper limb 18 hours before the operation itself. Patients randomized in the L-RIPC group had a significantly lower serum level of troponin I in the eight hours after surgery [66]. While the second study published so far and conducted in children did not find differences in serum levels of troponin I, the L-RIPC group had lower levels of NT-BNP (N terminal pro-B-type natriuretic peptide) [89].

## 6. Pharmacological Cardioprotection

Revelation of the principles of ischaemia-reperfusion myocardial injury, on the one hand, and congenital defence mechanisms, on the other hand, offers the possibility of pharmacological intervention. Cardioprotective agents may be applied before application of the aortic clamp, added to the cardioplegic solution, or used in the reperfusion stage, or a combination of these may be an option. Just as with STEMI patients, drug administration may be considered during the acute myocardial ischaemia or in the reperfusion stage. The experiment successfully tested many substances and processes, clinical testing reducing the number of positive ones, and the most promising are now being tested in large sets of patients in multicenter studies. Recently, drugs are being tested (and attracting great attention) that mimic ischaemic conditioning (IC), at all of the three hierarchical levels, adenosine being amongst the first of such pharmaceuticals, the substance ranking among the IC triggers. For CABG patients, the administration of adenosine (intravenously or as part of cardioplegia) was associated with less myocardial injury and faster postoperative recovery [90, 91]; other studies were less conclusive [92, 93] and any further testing in cardiac surgery was paused due to hypotensive side effects. Similar side effects were exhibited by bradykinin [94]. Our research group tested tramadol on CABG patients, an opioid that also shows the serotonin effect, for which cardioprotective effects were demonstrated as well [95, 96]. Conversely, however, in this study tramadol increased postoperative serum levels of troponin I quite significantly. The explanation lies in a possible paradoxical serotonin response in patients with coronary artery disease. Serotonin dilates normal coronary arteries, while in atherosclerotic arteries it causes vasoconstriction [97]. Another substance that activates intracellular defensive pathways is atrial natriuretic peptide. Infusion of carperitide, the synthetic analogue, when started after primary PCI reduced infarction size in STEMI patients, as measured by lower levels of cardiac enzymes (15% reduction) and maintaining systolic function measured by EFLK [98]. Exenatide, a new antidiabetic drug with cardioprotective properties, appears to be more promising because it has demonstrated efficiency even over longer follow-up periods. The infusion of exenatide initiated 15 minutes prior to primary PCI in STEMI patients decreased infarction size by 23% as documented using CMRI (cardiac magnetic resonance imaging) 90 days after the intervention [99]. Inhaled anaesthetics are a group of agents which have shown cardioprotective effects by influencing multiple hierarchical pathways. Meta-analysis of 27 clinical trials in CABG patients described in the sevoflurane group a lower release of troponin, less inotropic support, and preserved ventricular function [100]. Sevoflurane testing is now underway in acute cardiology in STEMI patients subjected to reperfusion (SIAM trial: In terms of mechanism of action, great attention is paid to cyclosporine A, which inhibits opening of MPTP channels in the mitochondria in the post-ischaemia reperfusion stage). Complex beneficial effects of nitric oxide donors in cardioprotection were described, which may be summarized in three points: (1) a direct haemodynamic effect mediated through vasodilation of coronary arteries, (2) a direct effect on improving cardiac output, and (3) an increase in vascular sensitivity to sympathetic stimulation could lead to increased diastolic blood pressure [101]. NO can also directly modify sulfhydryl residues of proteins through S-nitrosylation, which has emerged as an important posttranslational protein modification. S-nitrosylation of critical protein thiols has been shown to protect them from further oxidative modification by reactive oxygen species. Recently it has been suggested that S-Nitrosylation could play important role in cardioprotection [102]. Cyclosporine thus intervenes at the end-effector, where the defensive signals of intracellular pathways converge. A small clinical study on 27 STEMI patients treated by PPCI demonstrated in the cyclosporine group a 20% reduction in infarction on CMRI [103]. This and another study, which observed a lasting effect even after six months on the CMRI, gave rise to a wider study of CIRCUS trial: NCT01502774, which is now underway [104]. Other more specific inhibitors of MPTP channels are now being tested in ongoing trials (MITOCARE: NCT01374321, EMBRACE: NCT01572909) [105, 106]. Further interventions and pharmaceuticals that appeared to be efficient in animal models are being tested in acute cardiology: mecasermin, insulin-like growth factor analogue (RESUS-AMI: NCT01438086) [107], mangafodipir, iron oxidation inhibitor and chelator (MANAMI: NCT00966563) [108], melatonin (MARIA: NCT00640094) [109], inhaled nitric oxide (NOMI: NCT01398384) [110], IV sodium nitrite (NIAMI: NCT01388504) [111], intracoronary sodium nitrite (NITRITE-AMI: NCT01584453) [112], thymosin beta-4, growth regulator (NCT00378352) [113], and metoprolol (METOCARD-CNIC: NCT01311700) [114]. Those ongoing studies are summarized in Table 1. On the other hand, a number of pharmaceuticals proved inefficient in laboratory trials. In CABG patients, failure was found in cariporide, sodium-hydrogen exchanger inhibitor (GUARDIAN and EXPEDITION trials) [115, 116], acadesine, adenosine precursor (RED-CABG trial) [117], pexelizumab and C5 complement inhibitor (PRIMO-CABG trial) [118], while in STEMI patients failure was found in trimetazidine, fatty acid oxidation inhibition (EMIP-FR 2000 trial) [119], eniporid, sodium-hydrogen exchanger inhibitor (ESCAMI trial) [120], delcasertib, protein kinase C inhibitor (PROTECTION-AMI) [121], atorvastatin (REPARATOR trial) [122], and also magnesium (MAGIC trial) [123] and glucose-insulin-potassium infusion (CREATE-ECLA) [124]. According to the results of the REVEAL study, in patients with STEMI who had successful reperfusion with primary or rescue PCI, a single intravenous bolus of epoetin alfa within four hours of PCI did not reduce infarct size and was associated with higher rates of adverse cardiovascular events [125]. Moreover, erythropoietin may increase clinical adverse events [125, 126]. FX06, a naturally occurring peptide derived from human fibrin, has been shown to reduce myocardial infarct size in animal models by mitigating reperfusion injury. On the other hand in the human FIRE Trial, FX06 reduced the necrotic core zone as one measure of infarct size on magnetic resonance imaging, while total late enhancement was not significantly different between groups [127].

## 7. Challenges and Perspectives in Translation to Clinical Outcomes

The above studies represent only a small part of the efforts and resources that have been expended in this field. In the US alone, it is estimated that over the last 40 years, hundreds of millions of dollars were spent in preclinical studies for so-called infarction life-saving therapies. This gave rise to hundreds of treatments that were identified as controlling myocardial infarction. Nonetheless, these enormous resources have failed to lead to clinical application due to a number of methodological shortcomings.

Mechanisms of cellular defensive response to ischaemic stress and pharmacological interventions were investigated using animal, tissue, and subcellular models, particularly in mice, rats, and rabbits, and, to the lesser extent, large animals. Thus, they cannot easily be transferred to human research, where differences may exist. In addition, little is known about the spatial and temporal organisation of these defence mechanisms. This may be one of the reasons for pharmacological cardioprotection failing in clinical trials [128]. Pharmacological influence of the reperfusion injury runs into the issue of targeted application in effective concentrations. Solutions may include the drug to be enclosed in liposome nanoparticles, since nanoparticles preferentially accumulate at sites with increased postischaemia vascular permeability. For example, adenosine encapsulated in nanoliposomes boosts local cardioprotective effects without evidence of systemic hypotension [129].

In 2003, a workshop took place in the US, initiated by NHLBI (National Heart, Lung, and Blood Institute), the event's title, “Transfer of Therapies to Protect the Myocardium from Ischemia,” was self-explanatory and its main recommendations included continuation of the clinical testing of adenosine [130]. The AMISTAT 2 trial of 2005 compared infarction size in STEMI patients treated with PPCI with three-hour infusion of adenosine with dual concentration: 50 and 70 *μ*g/kg/minute versus placebo. SPECT (single photon emission CT) revealed the smallest infarction in higher concentrations: 11%, 23% versus 27% for placebo [131]. However, the second NHLBI workshop in 2010 still identified a number of gaps existing in the knowledge of the key moments of acute reperfusion injury and defensive response of the organism. The mechanism of lethal reperfusion injury, as well as its possible influence, is still not explained. The same applies to the exact mechanism of microvascular obstruction (no-reflow phenomenon). There is also no determination regarding which cardioprotective therapy may be appropriate in which clinical situations. The so-called combination therapies, which could strengthen the efficiency of individual measures, were not tested to a great extent. As cardioprotective therapies fail in the patients who need them most, that is, older persons and persons with comorbidities (diabetes, hypertension, and dyslipoproteinaemia), it will therefore be necessary to find procedures that work especially in this regard. Finally, there is the necessity of identifying molecular markers that would indicate the presence of the cardioprotective state [132]. Challenges prevail over resolved issues; preclinical studies are, however, a good start. In 2011, the NHLBI awarded a five-year grant to the initiative called the Consortium for Preclinical Assessment of Cardioprotective. Therapies (CAESAR), which will test promising cardioprotective therapies through the application of standard and randomized protocols carried out by blinded researchers and analysed by blinded analysis, as with clinical studies. The main aim of this consortium is to ensure repeatability of the results on relevant animal models, including conscious animals and comorbid models [133]. Optimism prevails in Europe as well. In 2013, a working group for cell biology of the heart, attached to the European Society of Cardiology, published a document entitled “Transferring Cardioprotection for the Benefit of the Patient,” its main proposition comprising the belief that the failure was in the inability successfully to transfer promising therapies into procedures that improve clinical outcomes, rather than in a lack of potential cardioprotective therapies in preclinical research [134].

## 8. Conclusion

The fundamental discovery of cardioplegic myocardial protection in cardiac surgery in the 1970s and 1980s enabled effective surgical treatment bringing prolonged and better-quality life to the patient. Also, early reperfusion in STEMI patients remains the only effective treatment in cardiology. Both therapeutic strategies, however, are accompanied by reperfusion injury of the myocardium, which leads to dysfunction and/or loss of part of the myocardium. During the same period, another fundamental discovery was made: termed ischaemic preconditioning, it activates protection from ischaemia-reperfusion myocardial injury. The extensive research on this phenomenon in the past three decades has revealed basic mechanisms and suggested methods of use. Thus, the question is whether activation or augmentation of the defence mechanism may enhance myocardial protection in cardiac surgery or rescue another portion of the jeopardised myocardium after reperfusion therapy in STEMI patients. Although widespread, the evolutionary young cellular mechanisms of protection against oxygen handling are not very robust. Ischaemic conditioning, which is among them, is also thus limited and for myocardium, the organ with the highest oxygen turnover, it is of undeniable importance. At present, the prevailing belief is that such options of treatment exist for reperfusion myocardial injury, but the time for their full employment has not yet come, due to unresolved subquestions and methodological issues with the transfer into clinical practice.

## Figures and Tables

**Table 1:** Overview of ongoing major clinical studies in pharmacological cardioprotection.

Agent	Property	Clinical trial	Reference
Cyclosporine	mPTP inhibitor	CIRCUS trial: NCT01502774	[103]
TRO40303	mPTP inhibitor	MITOCARE: NCT01374321	[104]
Bendavia	mPTP inhibitor	EMBRACE: NCT01572909	[105]
Mecasermin	IGF analogue	RESUS-AMI: NCT01438086	[106]
Mangafodipir	Iron oxidation inhibitor and chelator	MANAMI: NCT00966563	[107]
Melatonin	Multimodal effects	MARIA: NCT00640094	[108]
Inhaled Nitric Oxide	Vasodilator, mPTP inhibitor	NOMI: NCT01398384	[109]
IV sodium nitrite	Vasodilator, mPTP inhibitor	NIAMI: NCT01388504	[110]
Intracoronary sodium nitrite	Vasodilator, mPTP inhibitor	NITRITE-AMI: NCT01584453	[111]
Thymosin beta-4	Growth regulator	NCT00378352	[112]
Metoprolol	*β*-blocker	METOCARD-CNIC: NCT01311700	[113]
